# Supplementary material for: Fabrication of Dragee Containing Spirulina platensis Microalgae to Enrich Corn Snack and Evaluate Its Sensorial, Physicochemical and Nutritional Properties
Source: Foods. 2022 Jun 27;11(13):1909. doi: 10.3390/foods11131909 (PMC9265436; doi:10.3390/foods11131909)
Supplement: Supplementary file 1 [file foods-11-01909-s001.zip › foods-1753824-supplementary.pdf]

foods
MDPI

Supplementary Materials

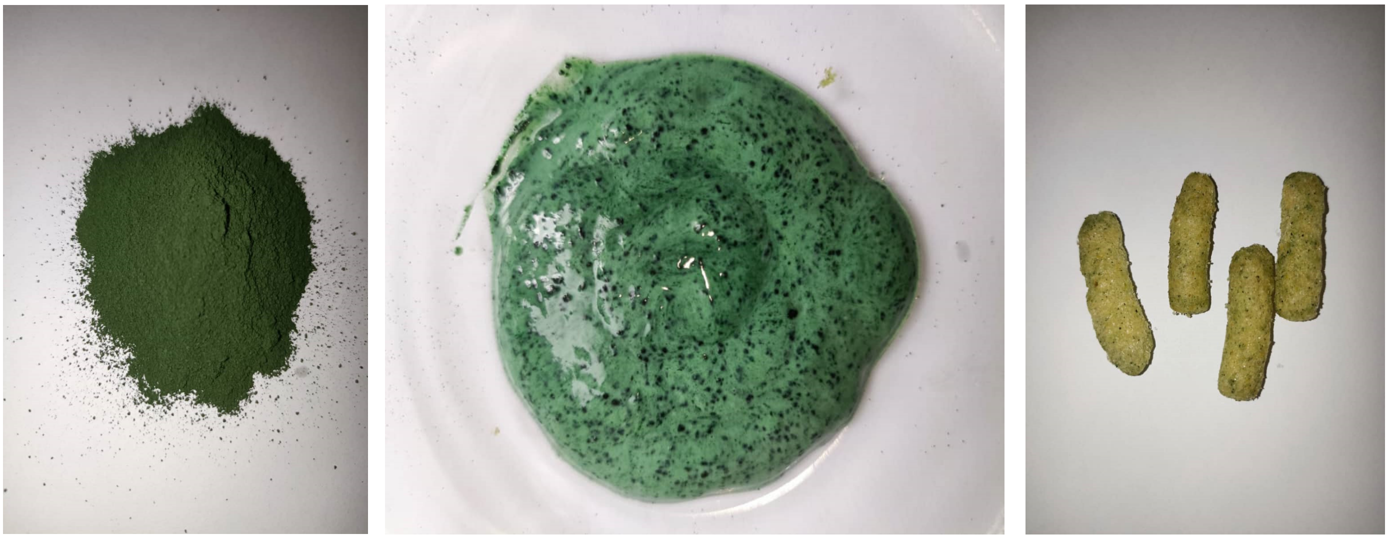

SP powder	Dragee containing SP	Snack with dragee containing SP

Figure S1. SP algae powder, dragees containing SP, and snacks coated with dragees containing SP.
